# Fetuin-A and Heme Oxygenase 1 as Potential New Markers in the Diagnosis of Diabetic Kidney Disease

**DOI:** 10.3390/ijms26209862

**Published:** 2025-10-10

**Authors:** Magdalena Król-Kulikowska, Agata Przyborska, Emilia Miernikiewicz, Patrycja Roszykiewicz, Wiktoria Roszykiewicz, Mirosław Banasik, Marta Kepinska

**Affiliations:** 1 Department of Pharmaceutical Biochemistry, Faculty of Pharmacy, Wroclaw Medical University, Borowska 211a, 50-556 Wroclaw, Poland; 2 Students’ Scientific Association at the Department of Pharmaceutical Biochemistry (SKN No. 214), Faculty of Pharmacy, Wroclaw Medical University, Borowska 211A, 50-556 Wroclaw, Poland; agata.przyborska@student.umw.edu.pl (A.P.); emilia.miernikiewicz@student.umw.edu.pl (E.M.); patrycja.roszykiewicz@student.umw.edu.pl (P.R.); wiktoria.roszykiewicz@student.umw.edu.pl (W.R.); 3 Department and Clinic of Nephrology and Transplantation Medicine, Faculty of Medicine, Wroclaw Medical University, Borowska 213, 50-556 Wroclaw, Poland; miroslaw.banasik@umw.edu.pl

**Keywords:** fetuin-A, heme oxygenase 1, diabetic kidney disease, single-nucleotide polymorphism, biomarker

## Abstract

Due to their prevalence, diabetes and its complications continue to pose a significant challenge in modern medicine. It is particularly important to identify and develop new biomarkers that would enable faster and more effective detection of specific diseases, including the most common complication of diabetes—diabetic kidney disease (DKD). This review presents the current knowledge on two proteins—fetuin-A and heme oxygenase 1 (HO-1)—whose biological functions and involvement in the pathophysiology of the discussed disease make them potentially useful biomarkers. Moreover, there are studies indicating an association of polymorphisms in the genes encoding fetuin-A and HO-1 with the risk of developing diabetes or DKD. Based on the available literature, both proteins appear promising for use in the diagnosis of diabetes and its complications or assessing the risk of these diseases. However, this requires confirmation in large-scale studies and the development and standardization of detection methods.

## 1. Introduction

Diabetes is a real challenge for modern medicine. Due to its prevalence and lifestyle factors, it is considered a lifestyle disease [1]. Left untreated, it can lead to serious complications and significantly reduce quality of life, even shortening it. One of the most common and serious complications of diabetes is diabetic kidney disease (DKD), also known as diabetic nephropathy (DN), which leads to end-stage renal disease (ESRD) and increased mortality from cardiovascular disease [1,2]. Early detection of changes in the kidneys is crucial for halting disease progression and implementing effective therapy. Currently used parameters, such as creatinine concentration, albuminuria/proteinuria assessment, and evaluation of renal filtration function—reflect kidney damage only at more advanced stages. Therefore, the search for new markers that would allow for the early detection of DKD is of critical importance crucial [3,4].

One such potential biomarker is fetuin-A, a glycoprotein primarily expressed in the liver and adipose tissue [5,6]. Fetuin-A is responsible for transporting free fatty acids in the blood and is also involved in the regulation of inflammatory processes, mineralization, and insulin metabolism [7,8,9]. Its increased concentrations are observed in various metabolic conditions, including diabetes and DKD [10,11].

Another important parameter is heme oxygenase 1 (HO-1), an enzyme with potent cytoprotective and antioxidant properties. It catalyzes the degradation of heme to biliverdin or bilirubin, ferrous ions, and carbon monoxide. Under homeostatic conditions, its concentration is virtually undetectable. However, renal HO-1 transcription increases under the influence of various factors (oxidative stress, hypoxia, heavy metals, toxins) [12,13]. HO-1 plays a role in the cellular response to oxidative stress, which is a key factor in the pathogenesis of DKD [14]; therefore, this enzyme appears to be a potentially good prognostic marker.

In the public consciousness, the pathophysiology of diabetes and DKD is still primarily associated with an unhealthy diet and lack of exercise. However, genetic factors also play a role in the development of these diseases. With this in mind, it is also worth examining the impact of polymorphisms of the genes encoding fetuin-A and HO-1 on the risk of developing diabetes and its complications. In many studies, the polymorphism of the gene encoding fetuin-A (*AHSG*)—the rs4917 polymorphism—also appears to be associated with kidney damage or disease [15,16]. In turn, studies on the impact of polymorphisms of the gene encoding HO-1 also seem to indicate a potential role of the rs2071746 polymorphism in assessing the development of kidney disease [17,18]. These examples demonstrate the potential role that polymorphisms of the genes encoding fetuin-A and HO-1 may play in the risk of developing or progressing DKD. Based on genotypic variants within two selected polymorphisms and concentrations of potential biomarkers (fetuin-A and HO-1), a predictive model can be developed for faster diagnosis of DKD.

The aim of this article is to review the current state of knowledge and available data on the potential role of fetuin-A and HO-1 as novel biomarkers in the diagnosis of DKD. Particular emphasis is placed on their structure, mechanisms of action, and involvement in the pathogenesis of DKD, as well as the potential clinical applications of their measurements.

## 2. Diabetic Kidney Disease—Etiology, Pathogenesis, and Diagnostics

### 2.1. Etiology and Pathogenesis of Diabetic Kidney Disease

The etiology and pathogenesis of the development and progression of DKD are complex and multifactorial. The development of the disease is influenced by both hemodynamic abnormalities and metabolic disorders [19]. Hyperglycemia, proteinuria, lipid overload, and accumulation of advanced glycation end products are factors leading, among others, to kidney damage in patients with diabetes [20,21,22,23]. Hyperglycemia contributes to the deposition of extracellular matrix components and leads to a serious condition such as renal fibrosis [24,25].

Oxidative stress plays an important role in the pathogenesis of DKD. Oxidative stress induced by hyperglycemia leads to increased levels of proinflammatory proteins through the infiltration of macrophages, which secrete inflammatory cytokines, resulting in inflammation [26]. It can also lead to direct damage to podocytes, mesangial cells, and endothelial cells, leading to tubulointerstitial fibrosis [27,28]. Oxidative stress, which is induced by chronic hyperglycemia, may also result in increased levels of angiotensin II (Ang-II), which activates transforming growth factor-β (TGF-β) and stimulates mesangial matrix synthesis. TGF-β is involved in the production of reactive oxygen species (ROS), which is mediated by reduced nicotinamide adenine dinucleotide phosphate (NADPH) oxidase. Continuous activation of TGF-β leads to excessive remodelling of the extracellular matrix in the mesangium and contributes to fibrosis in the tubular interstitium [29].

Research shows that Ang-II affects the kidneys through systemic action as well as local activation of renin–angiotensin system (RAS) in the kidneys, which allows us to state that Ang-II is a molecule that plays an important role in physiological and pathological mechanisms of the kidneys [27,28]. It was found that in DKD, an increase in intrarenal Ang II level is observed, which leads to the activation of immune cells and the production of chemokines [30]. Intrarenal Ang-II may contribute to progressive renal damage through mechanisms such as increased glomerular capillary pressure and permeability leading to proteinuria, as well as renal cell hypertrophy, stimulation of macrophage infiltration, and inflammation [31,32]. Many studies have shown that Ang-II blockade may be beneficial in slowing disease progression [33,34].

Signalling cascades and immune cells activated by oxidative stress further promote inflammation [35]. The sustained chronic inflammation contributes to the kidney tissue remodelling, leading to fibrosis and further loss of function [31,32,36,37]. Inflammation may result from biochemical, metabolic, and hemodynamic disorders present in DKD [38].

Immune cells involved in the pathogenesis of DKD are mainly leukocytes, monocytes and macrophages. Increased macrophage infiltration in the glomeruli was observed in the patients’ renal biopsies [39]. Clinical studies also show that during the early development of DKD in patients, T lymphocytes and the tumour necrosis factor α (TNF-α) signalling pathway are activated [40,41]. Inflammatory factors such as IL-6, TNF-α, TGF-β 1 and IL-18 also play a key role in the development and progression of DKD [42]. According to studies, the level of circulating IL-6 positively correlates with the progression of DKD [43], while IL-1β, IL-18 and IL17A are associated with the occurrence and development of DKD [44,45,46]. Increased expression of chemoattractant cytokines and adhesion molecules, which are important mediators of kidney damage, is observed in diabetic animals and in renal cells of diabetic patients [19].

**Figure 1:**
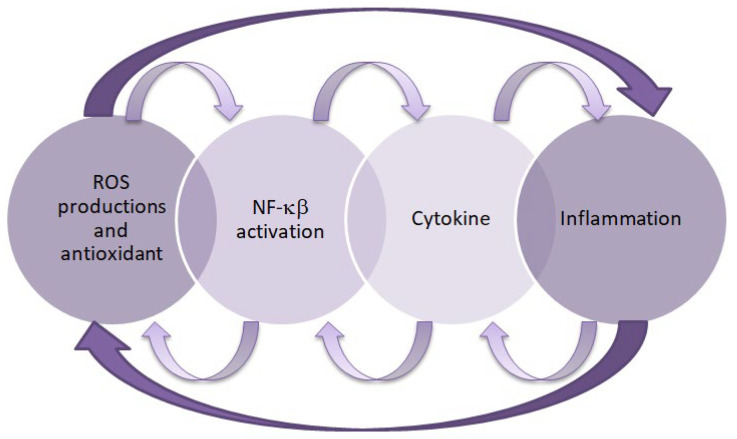
Relationship between inflammation and oxidative stress. This figure was created based on [50]. ROS—reactive oxygen species, NF-κB—nuclear factor kappa B.

Inflammation and oxidative stress are interdependent pathophysiological processes, as confirmed by research [47,48]. Oxidative stress activates nuclear factor kappa B (NF-κB), which regulates the expression of proinflammatory genes, while proinflammatory cytokines stimulate enzymes that generate free radicals. When oxidative stress occurs, inflammation develops and further amplify oxidative damage. Conversely, primary inflammation can induce oxidative stress, thereby perpetuating and intensifying the inflammatory response [49]. Therefore, both of these processes play an important role in the pathogenesis of DKD. These relationships are shown in Figure 1.

### 2.2. Diagnostics of Diabetic Kidney Disease

The diagnosis of DKD is based on criteria such as deterioration of renal function, the presence of proteinuria and diabetic retinopathy, as well as a decrease in glomerular filtration rate (GFR). One of the diagnostic indicators of DKD is persistent albuminuria with concomitant diabetic retinopathy and the absence of other kidney diseases [51,52]. Albuminuria can be measured using the ACR index, which is the ratio of albumin concentration in mg to creatinine concentration in g in a morning urine sample or during a 24 h urine collection [51]. Table 1 presents the classification of albumin concentration in urine based on the Kidney Disease: Improving Global Outcomes (KDIGO) 2024 Clinical Practice Guideline for the Evaluation and Management of Chronic Kidney Disease.

Prognostic based on albuminuria is not specific for DKD, because some patients with DKD do not have albuminuria [54,55,56].

According to the recommendations of the American Diabetes Association, screening for DKD in patients with type 2 diabetes (T2D) should be performed at the time of diagnosis and then annually. In the case of patients with type 1 diabetes, albuminuria tests should be performed five years after diagnosis and then annually. If an increase in albuminuria is detected in the patient, the tests should be repeated within three to six months. Increased albuminuria in a given person can be confirmed if at least two of three ACR tests indicate abnormal urine albumin concentration. Such patients should also be further examined to check for the presence of comorbidities [57].

To assess renal function, serum creatinine concentration is often measured and estimated glomerular filtration rate (eGFR) is calculated based on this concentration. However, creatinine measurement is susceptible to certain interferences resulting from the dependence of this marker on muscle mass, diet, tubular secretion and comorbidities such as advanced liver disease [58].

There are various formulas for eGFR, but they do not directly measure renal tissue damage and are also poorly sensitive to small changes in renal function. Differences in eGFR formulas based on serum creatinine levels further limit its usefulness [59,60,61]. GFR can also be estimated from serum cystatin C, which is a low molecular weight protein and has less interindividual variability due to its lack of dependence on muscle mass, age, gender, and inflammatory conditions. In addition, increased urinary cystatin C excretion, suggesting tubular damage, is observed in early and prediabetic nephropathy [62,63]. Table 2 presents a comparison between cystatin C and creatinine.

Renal biopsy is a rarely performed test in the diagnosis of DKD. It is invasive and carries potential risks. It is resource-intensive (time, cost, expertise) and may not be appropriate for older patients or those with comorbidities. However, it helps to make a definitive diagnosis and identifies atypical features [64]. There are four classes of histological changes characteristic of DKD. Class I presents with mild or nonspecific changes on light microscopy and accompanying thickening of the glomerular basement membrane. Class IIa presents with mild, while class IIb presents with severe mesangial expansion. Class III is characterized by the presence of nodular sclerosis (Kimmelstiel–Wilson changes), while class IV presents with advanced glomerulosclerosis [65].

## 3. The Role of Fetuin-A and AHSG Polymorphisms in the Pathogenesis of Diabetic Kidney Disease

### 3.1. Structure and Functions of Fetuin-A

Fetuin-A (also known as α2-Heremans-Schmid glycoprotein) is a multifunctional serum protein that belongs to the cystatin superfamily [66]. The *AHSG* gene is located on the 3q27 locus [67] and contains seven exons and six introns [68] encoding the protein. Transcription of this gene yields a single mRNA transcript that encodes a 367-amino acid single-chain preprotein of human fetuin-A. Following translation, fetuin-A undergoes a series of post-translational modifications, including glycosylation, proteolytic cleavage, proper folding, and phosphorylation, which are essential for the production of its biologically active form [10]. Mature fetuin-A consists of two polypeptide chains connected by a disulfide bond [69], forming three domains: two cystatin domains D1 and D2, and a C-terminal specific region [70].

Scientific reports indicate that fetuin-A is a multifunctional protein that plays a number of important roles in the human body [5,7,9]. Due to its high expression and significant capacity for molecular interactions with several different ligands, it is believed that fetuin-A, similar to albumin, may perform maintenance and clearance functions, participating in numerous physiological processes, including the regulation of metabolism, tissue mineralization, and inflammation [71].

Fetuin-A is believed to be responsible for insulin signalling, endocytosis, brain development, and protein metabolism [72]. It inhibits insulin receptor activity, thereby reducing tyrosine phosphorylation, which results in reduced tissue sensitivity to insulin. Therefore, it has also been linked to the pathophysiology of many metabolic disorders, including insulin resistance, diabetes, and its complications [73]. Elevated fetuin-A levels are observed in patients with visceral obesity, T2D, and metabolic syndrome [74]. Although it is synthesized primarily in the liver, small amounts are also produced in adipose tissue and other metabolically active cells. This allows it to act as an adipokine-like protein, influencing energy metabolism and inflammatory processes, leading to the development of insulin resistance. By binding to the toll-like receptor 4 (TLR4) on adipocytes and macrophages, fetuin-A activates proinflammatory signalling pathways, leading to the release of proinflammatory cytokines [75].

Fetuin-A also plays a crucial role in regulating bone remodelling (osteogenesis and bone resorption) and calcium metabolism [10]. By binding to calcium ions and phosphates, it forms colloidal complexes—calciprotectins—that prevent the spontaneous precipitation of calcium-phosphate salts in human plasma. Fetuin-A thus protects the body against pathological calcium deposition. Patients with fetuin-A deficiency experience increased vascular calcification [76].

Interestingly, fetuin-A has been shown to possess both anti-inflammatory and inflammatory properties, participating in neutrophil and platelet degranulation, lymphocyte stimulation, keratinocyte migration, and transporting metals and small molecules in the blood by binding to fatty acids, thyroid hormones, phosphates, and calcium ions [77,78]. In inflammatory processes, fetuin-A can act as either a stimulating or suppressive protein, depending on the stimuli. During inflammation, proinflammatory cytokines involved in the early phase, such as TNF-α, interleukins (IL-1, IL-6) and interferon gamma (IFN-γ), are susceptible to inhibiting fetuin-A expression, thereby exacerbating the inflammatory response and resulting excessive accumulation of late mediators. Fetuin-A has also been reported to closely interact with immune cells and exhibit potent opsonin functionality toward cationic spermine, making it a key element in the regulation of the innate immune response [5].

In addition, fetuin-A is also known to be a negative acute-phase protein, as it is often negatively correlated with CRP and IL-6 concentrations [9,79]. Fetuin-A acts as a direct antagonist to TGF-β and tumour necrosis factor-alpha (TNF-α) mediated inflammation, as well as inhibits the release of high mobility group box protein 1 (HMGBP1) by innate immune cells in response to pathogen-associated molecular patterns (PAMPs) [9].

Although there are not many studies directly connecting DKD and fetuin-A concentration, there are presumptions that a relationship may exist. Numerous studies associate elevated levels of fetuin-A with a higher risk of developing type 2 diabetes mellitus [80,81,82,83]. This statement is further supported by the mechanism by which fetuin-A affects insulin signalling, thereby having an impact on glycemia. Fetuin-A serves as a natural inhibitor of the insulin receptor, specifically the receptor tyrosine kinase (RTK). By interacting with the RTK, fetuin-A effectively disrupts the intracellular insulin signalling pathway, preventing the autophosphorylation of both the tyrosine kinase and insulin receptor substrate-1 (IRS-1) [9].

It is also worth noting that studies have demonstrated that in patients with chronic kidney disease, serum fetuin-A concentrations progressively decline in parallel with the deterioration of renal function, as assessed primarily by estimated glomerular filtration rate (eGFR) [84,85]. Based on previous research it may be hypothesized that fetuin-A could serve as a potential biomarker for the early detection of DKD.

### 3.2. Biosynthesis and Bioregulation of Fetuin-A

Transcription of *AHSG* produces a single copy of the mRNA that encodes the single-chain human fetuin-A preprotein, containing 367 amino acids. The transcription process is regulated by several CCAAT (C/EBP)-β enhancer and nuclear factor (NF)-1 binding sites in the region. The nascent prefetuin-A consists of two polypeptide chains: the A heavy chain (282 amino acid residues) and the B light chain (27 amino acid residues). The precursor protein also contains an additional 18-amino acid signal sequence (SS) at the N terminus and a 40-amino acid connecting peptide (CP) between the two chains. Active fetuin-A is produced by posttranslational modifications of the A and B polypeptide chains, which include glycosylation, proteolytic cleavage, folding, and phosphorylation. The modified A and B chains fold correctly and are held together by a disulfide bond, forming heterodimeric, three-dimensional mature glycoproteins [9,68].

Mature fetuin-A is released into the bloodstream from the liver, but this process is influenced by many factors. For example, elevated blood glucose and free fatty acids (FFA) levels contribute to increased fetuin-A release. High blood glucose levels induce its release by stimulating extracellular signal-regulated kinase 1/2, while elevated blood FFA levels promote its secretion by increasing NF-κB activity [86]. Furthermore, external factors can also influence the bioregulation of fetuin-A, contributing to its reduced release and consequently lowering its blood concentration. Such factors include curcumin, resveratrol, dairy products, niacin, alcohol, and coffee [9]. Not all mechanisms of action of these products are well understood, but it is known that curcumin reduces fetuin-A expression by regulating peroxisome proliferator-activated receptor-γ (PPAR-γ) and stimulating AMP-activated kinase [87]. In turn, niacin has been shown to lower blood lipid levels, which negatively impact fetuin-A expression [88]. Conversely, dietary intake of omega-3 fatty acids has been found to increase fetuin-A secretion by the liver [89].

### 3.3. The Role of AHSG Polymorphisms in the Development of Selected Diseases

There are numerous scientific reports on the impact of polymorphisms in *AHSG* on the risk of developing selected diseases, including diabetes, kidney disease or cardiovascular disease. This review primarily discusses single-nucleotide polymorphisms (SNPs), which involves single-base substitutions [90,91,92]. For example, the rs4918 polymorphism was studied in the context of its influence on the glycosylation pattern of human serum fetuin-A. Lin et al. [93] assessed whether the rs4918 polymorphism affects the glycosylation of fetuin-A. One intense peak with a mass of 42,633.02 Da was detected in AHSG*2 and AHSG*1/2 (42,632.24 Da). The same peak was completely absent in all AHSG*1. Thr256 in the AHSG*1 peptide was fully occupied by O-glycans containing 0, 1, or 2 sialic acids, whereas Ser256 in the AHSG*2 peptide was mostly unmodified. Based on these results, it was concluded that the rs4918 polymorphism affects the glycosylation profile of fetuin-A, which can affect its function; however, the clinical significance of these changes requires further studies.

In 2017, the CHARGE consortium conducted a meta-analysis of genome-wide association studies to identify genetic loci associated with plasma fetuin-A [94]. Three SNPs stood out in this study: rs4917, rs4918, and rs1900618. Each additional copy of the T allele (rs4917 polymorphism) was associated with a decrease in fetuin-A levels of approximately 0.066 ± 0.002 g/L in each cohort. The rs4917 polymorphism explained 14% of the variance in fetuin-A levels among European Americans. Genome-wide association analyses for rs4917 revealed that approximately 40 polymorphisms (SNPs) in the *AHSG* locus were associated with fetuin-A levels. The main genetic determinants of fetuin-A levels were located in the gene encoding fetuin-A. The SNPs with the strongest association in both ethnic groups were not identical (rs4917 in European Americans and rs1900618 in African Americans). Genetic variation in *AHSG* was found to be strongly associated with fetuin-A levels, particularly for the rs4917, rs4918, and rs1900618 polymorphisms.

In turn, another study examined the association of selected SNPs in *AHSG*—rs4917 and rs4918—with serum fetuin-A levels, coronary artery calcification (CAC), and mortality in patients after kidney transplantation, and in patients with chronic kidney disease (CKD) [16]. The TT genotype (rs4917 polymorphism) and the GG genotype (rs4918 polymorphism) were associated with lower serum fetuin-A levels than the other genotypes. Serum fetuin-A levels were inversely correlated with each additional minor allele of the genotype (mutated T allele in the rs4917 polymorphism and G allele in the rs4918 polymorphism). Furthermore, lower serum fetuin-A levels correlated significantly with higher inflammatory parameters: fibrinogen, C-reactive protein (CRP), transferrin. This study confirmed that the rs4917 and rs4918 polymorphisms were associated with low serum fetuin-A levels in patients after radiotherapy and CKD [95].

Al Ali et al. [96] conducted a study to examine the association of genetically predicted fetuin-A with T2D and cardiovascular outcomes, including coronary artery disease, myocardial infarction, any stroke, and ischemic stroke. Genetically predicted fetuin-A was used because direct measurements of its plasma concentration were not available in the UK Biobank. A series of two-sample Mendelian randomized analyses were performed to assess whether genetically predicted fetuin-A was associated with cardiovascular traits and T2D. No association was found between genetically predicted fetuin-A and coronary heart disease, stroke, and ischemic stroke (patients with or without T2D). However, a one-standard deviation increase in genetically predicted fetuin-A increased the risk of T2D, but in a leave-one-out analysis, this was no longer significant if the rs11017848 polymorphism was excluded.

Other studies have also been devoted to assessing the role of *AHSG* polymorphisms in the risk of developing diabetes. A Mendelian randomization analysis was used to examine the association between circulating fetuin-A and the risk of developing T2D [97]. Data came from the EPIC-InterAct study and the DIAGRAM consortium and concerned five polymorphisms: rs4917, rs2070635, rs2070633, rs2248690, rs4831. Four of the five *AHSG* polymorphisms (excluding rs4831) were significantly associated with fetuin-A levels, and their increase was observed for the following alleles: C (rs4917), G (rs2070635), C (rs2070633), and A (rs2248690). It was therefore shown that the studied *AHSG* polymorphisms correlated with the level of fetuin-A. However, the level of fetuin-A did not correlate with the incidence of diabetes.

Moreover, an association between the rs4918 polymorphism in *AHSG* and the occurrence of gestational diabetes mellitus (GDM) has also been observed [98]. A significant difference was found between the frequency of the CC and GG genotypes within this polymorphism in women diagnosed with GDM. The rs4918 polymorphism involves a cytosine-to-guanine substitution and leads to a missense mutation, converting threonine to serine at position 256 of the synthesized protein, which affects its activity and levels. The GG variant may have a protective effect against the development of GDM, but this requires confirmation in large-scale studies. This study also assessed the effect of the rs2248690 polymorphism, but no statistically significant differences were observed in the genotype or allele distribution.

Fetuin-A also plays a role in patients with kidney disease. Studies have shown that it may act as a protective inhibitor of vascular calcification in patients with CKD and ESRD [99]. Morsy et al. [100] conducted a study to evaluate polymorphisms in *AHSG* (rs248 and rs256) and serum fetuin-A levels in patients with T2D and early DKD. In patients with T2D, the mean serum fetuin-A level was significantly higher compared with the control group, but fetuin-A levels did not correlate with eGFR or albuminuria. The genotype distribution of both polymorphisms showed a higher frequency of the TT genotype (rs248 polymorphism) and the GG genotype (rs256 polymorphism) among patients without albuminuria compared to those with microalbuminuria. Based on additional studies, elevated serum fetuin-A levels are associated with insulin resistance and an increased risk of atherosclerosis in patients with T2D, but not necessarily with the development of DKD. On the other hand, the TT (rs248 polymorphism) and GG (rs256 polymorphism) genotypes may be associated with a reduced risk of developing DKD.

Based on the accumulated evidence and comprehensive analyses, the rs4917 and rs4918 polymorphisms of the AHSG gene appear to have the greatest clinical relevance. The rs4918 (767C > G) variant results in a missense mutation leading to the substitution of threonine with serine at position 256 of the synthesized protein. This structural change alters not only the biological activity and circulating levels of fetuin-A but also its glycosylation profile, which may have downstream effects on protein stability and function [93,98]. Similarly, rs4917 represents a missense mutation with a well-documented impact on serum fetuin-A concentrations [94]. Taken together, these findings indicate that rs4917 and rs4918 polymorphisms may play a pivotal role in modulating fetuin-A biology, thereby contributing to their potential as clinically significant genetic determinants in the context of chronic kidney disease and related cardiovascular complications.

## 4. The Role of Heme Oxygenase 1 and HMOX1 Polymorphisms in the Pathogenesis of Diabetic Kidney Disease

### 4.1. Structure and Functions of Heme Oxygenase 1

**Figure 2:**
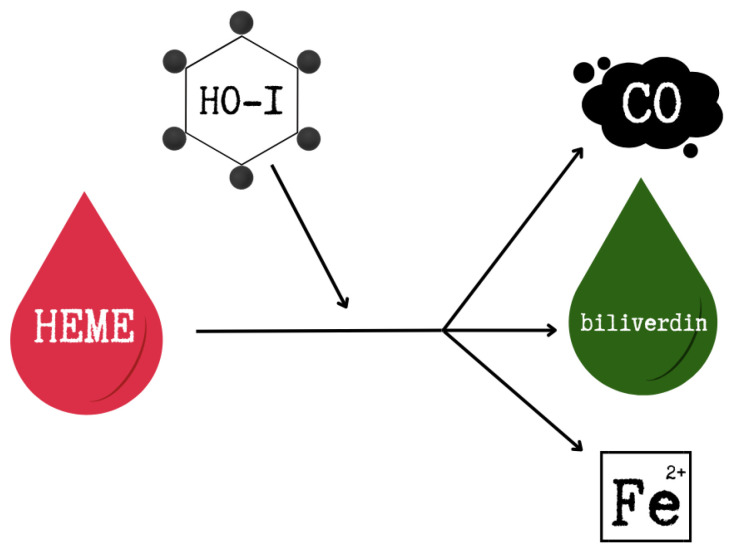
Heme degradation reaction. This figure was created based on [101]. HO-1—heme oxygenase-1, CO—carbon monoxide, Fe^2+^—free ferrous iron.

HO-1 is an essential enzyme responsible for the degradation of heme in the human body. This catabolic process leads to the breakdown of heme into carbon monoxide (CO), biliverdin (which is subsequently converted into bilirubin), and free ferrous iron (Fe^2+^), which is sequestered by ferritin to limit toxicity [101]. The degradation reaction requires molecular oxygen (O_2_) and NADPH. This reaction is shown in Figure 2.

*HMOX1* encoding HO-1 is located on chromosome 22q12.3 and spans approximately 13,148 base pairs. The human chromosome contains five exons and four introns. It encodes a 32 kDa protein that is typically expressed at low levels under physiological conditions. However, its expression is significantly upregulated in response to oxidative stress, inflammation, or disturbances in iron metabolism. HO-1 is predominantly expressed in tissues involved in heme turnover, such as the liver, spleen, and bone marrow. An additional element of the phenotype of the aforementioned polymorphism is the maintenance of redox homeostasis and the prevention of carcinogenesis, which is crucial in correlation with cancer cells [102].

**Figure 3:**
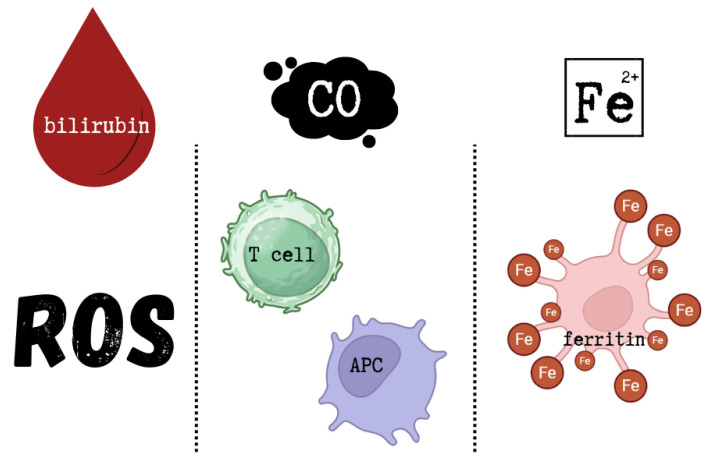
Cytoprotective effects of HO-1 metabolic products. This figure was created based on [102]. ROS—reactive oxygen species, CO—carbon monoxide, APC—antigen-presenting cells, Fe^2+^—free ferrous iron.

The cytoprotective effects of HO-1 are primarily attributed not to the enzyme itself, but to the biological activities of its metabolic products. Bilirubin acts as a powerful antioxidant, capable of scavenging ROS, while CO exerts anti-inflammatory effects by modulating T cells and antigen-presenting cells (APC). Free iron activates membrane Fe-ATPases, decreasing intracellular Fe^2+^ levels and thus reducing ROS formation. In addition, free iron induces the synthesis of ferritin, an iron-chelating protein that contributes to iron homeostasis [102]. The cytoprotective effects of HO-1 metabolic products are shown in Figure 3.

**Figure 4:**
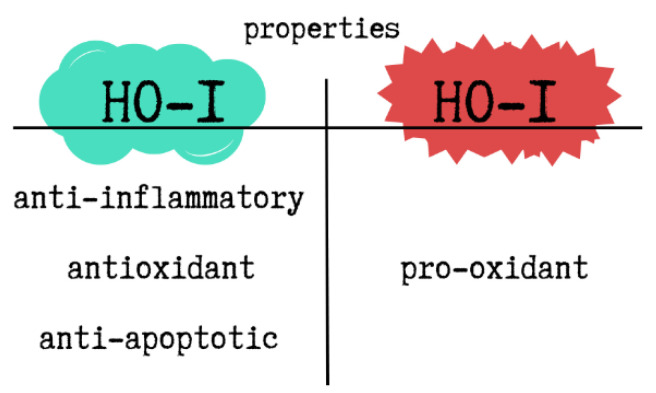
The dual properties of HO-1. This figure was created based on [102]. HO-1—heme oxygenase-1.

HO-1 has well-documented anti-inflammatory, antioxidant, and anti-apoptotic properties. It also regulates transcription, intracellular localization, and immunomodulatory pathways. As a pleiotropic molecule, HO-1 affects various signalling mechanisms, particularly under pathological conditions. However, depending on the cellular redox state and iron metabolism, HO-1 can also exhibit pro-oxidant effects. Excessive HO-1 expression has been shown to trigger ferroptosis—a form of programmed cell death dependent on iron-mediated lipid peroxidation. The entire process is based on free Fe^2+^, which comes from HO-1. Free Fe^2+^ induces the synthesis of the heavy chain of the iron-chelating protein ferritin and activates the membrane transporter Fe-ATPase. The result is a reduction in free Fe^2+^ concentration and a decrease in ROS production due to the Fenton reaction. However, in the case of excessive HO-1 activation, the excess free Fe^2+^ is not balanced by ferritin induction or quenching systems, which can result in ferroptosis. The accumulation of free Fe^2+^ leads to ROS overproduction and, consequently, cell death [102]. The dual properties of HO-1 are illustrated in Figure 4.

Numerous studies have highlighted that HO-1 deficiency may result in severe consequences, including anemia, chronic inflammation, vascular injury, nephropathy, asplenia, growth retardation, and tissue iron overload [103]. Conversely, elevated HO-1 expression contributes to cellular adaptation to stress and may have protective effects in multiple disease models.

In the cardiovascular system, HO-1 inhibits the proliferation and migration of vascular smooth muscle cells (SMCs), reducing intimal hyperplasia following arterial injury. This effect is mediated through the increased production of CO and bilirubin, which act to prevent vascular remodelling [104]. These properties suggest a potential therapeutic role for HO-1 in preventing atherosclerosis and other cardiovascular disorders.

Recently, increased attention has been given to polymorphisms in *HMOX1* and their influence on HO-1 expression. Certain polymorphic variants, particularly those leading to higher expression of HO-1, are considered protective against inflammation-related pathologies. Impaired transcription of *HMOX1*, on the other hand, may increase susceptibility to various diseases [105].

A well-characterized example involves a (GT)n microsatellite polymorphism in the *HMOX1* promoter region. The presence of a long allele in this region is associated with reduced HO-1 expression and a higher risk of developing rheumatoid arthritis and chronic obstructive pulmonary disease (COPD). In contrast, the short allele, which leads to increased expression of HO-1, appears to confer protection against these conditions [105].

### 4.2. Biosynthesis and Bioregulation of Heme Oxygenase 1

HO-1 is an enzyme with a molecular weight of approximately 32 kDa, abundantly present in tissues involved in the degradation of senescent erythrocytes, such as the liver, bone marrow, and spleen. The expression of HO-1 can be induced by a wide range of exogenous and endogenous stimuli, including hypoxia, hyperoxia, ultraviolet (UV) radiation, statins, heat shock, heavy metals, oxidative stress, inflammatory signals, cobalt protoporphyrin IX (CoPP), and iron deficiency [106].

HO-1 induction is mediated through several intracellular signalling pathways, primarily through the transcription nuclear factor erythroid 2–related factor 2 (Nrf2). These include mitogen-activated protein kinases (MAPKs), protein kinase C (PKC), AMP-activated protein kinase (AMPK), and the phosphoinositide 3-kinase/protein kinase B (PI3K/Akt) pathway. Additionally, stimuli such as hypoxia, oxidative stress, heme, and interleukin-6 (IL-6) promote HO-1 expression through the activation of various transcription factors including activator protein-1 (AP-1), signal transducer and activator of transcription 3 (STAT3), Yin Yang 1 (YY1), and hypoxia-inducible factor 1 (HIF-1) [105].

HO-1 is primarily localized to the endoplasmic reticulum, where it is anchored via its C-terminal (COOH) region, enabling close proximity to cytochrome P450 enzymes and efficient enzymatic activity. Its presence in other cellular compartments is often associated with COOH-terminal truncation and a corresponding loss of enzymatic function. HO-1 expression can be inhibited by BTB and CNC homology 1 (Bach1), as well as by metalloporphyrins such as zinc protoporphyrin IX (ZnPPIX) and tin protoporphyrin IX (SnPPIX), which compete with heme for binding to the enzyme’s active site [107].

Lipopolysaccharide (LPS), a potent inducer of oxidative stress, has been shown to upregulate HO-1 expression through AP-1 activation. The NF-κB subunits p50 and p65 also modulate HO-1 transcription. Oxidative stress, growth factors, proinflammatory cytokines, and IκB kinase activation lead to the ubiquitination and proteasomal degradation of IκB, allowing p50/p65 heterodimers to translocate to the nucleus and activate the HO-1 promoter. Under hypoxic conditions, HIF-1 serves as a key inducer of HO-1. MAPK signalling phosphorylates HIF-1α, promoting its nuclear accumulation and facilitating the formation of the HIF-1α/HIF-1β–p300/CBP complex, which binds to hypoxia-responsive elements in the HO-1 promoter and enhances transcription [108].

**Figure 5:**
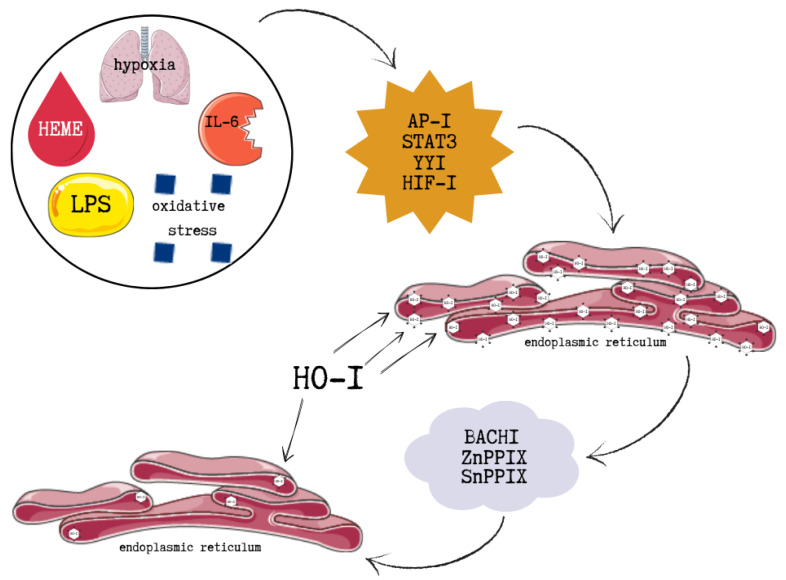
The molecular pathways illustrating positive and negative regulation of HO-1. This figure was created based on [103,108]. HO-1—heme oxygenase-1, LPS—lipopolysaccharide, IL-6—interleukin 6, AP-I—activator protein-1, STAT3—signal transducer and activator of transcription 3, YYI—Yin Yang 1, HIF-1—hypoxia-inducible factor 1, BACHI—BTB and CNC homology 1, ZnPPIX—zinc protoporphyrin IX, SnPPIX—tin protoporphyrin IX.

Nrf2 is the principal positive regulator of HO-1. Under basal conditions, it is sequestered in the cytoplasm by Kelch-like ECH-associated protein 1 (Keap1). ROS or electrophiles oxidize critical cysteine residues in Keap1, disrupting its interaction with Nrf2 and allowing newly synthesized Nrf2 to accumulate in the nucleus, where it activates HO-1 and other antioxidant genes. HO-1 transcription is also negatively regulated. Bach1 forms heterodimers with small Maf proteins and competes with Nrf2 for antioxidant response elements (AREs). Free heme binds Bach1, promoting its nuclear export and relieving transcriptional repression. Additionally, some BTB domain-containing proteins inhibit Nrf2’s DNA-binding activity, but heme-induced ROS counteract this effect by releasing Nrf2 from Keap1 and promoting its nuclear localization [103]. The above-mentioned pathways are illustrated in Figure 5.

Although oxidative stress is a major inducer of HO-1 expression, other signalling pathways and transcription factors also contribute to its regulation. These include pathogen-associated molecular patterns (PAMPs) and danger-associated molecular patterns (DAMPs). Several kinases involved in inflammation also modulate HO-1 expression, including tyrosine kinases, protein kinases A, B, C, and G, phosphatidylinositol 3-kinase (PI3K), and MAPK. MAPK has been shown to directly phosphorylate Nrf2, suggesting an indirect regulatory role, although it does not influence Nrf2’s binding to Keap1 [109].

### 4.3. The Role of HMOX1 Polymorphisms in the Development of Selected Diseases

#### 4.3.1. GT(n) Microsatellite Polymorphism

One of the most studied polymorphisms within the *HMOX1* promoter is the (GT)n dinucleotide repeat (rs3074372). The number of repeats typically ranges from 12 to 45, with a bimodal distribution in the general population, where alleles of approximately 23 and 30 repeats are most frequent [110].

Functionally, these repeats are categorized as short (S, <25 repeats) and long (L, ≥25 repeats). The S allele has been consistently associated with enhanced *HMOX1* transcriptional activity, as well as increased *HMOX1* mRNA expression levels compared to the L allele. Furthermore, lymphocytes isolated from carriers of the S allele display greater anti-apoptotic capacity [110].

In contrast, individuals carrying the L allele have demonstrated increased susceptibility to a range of diseases, including breast cancer, acute pancreatitis, acute kidney injury, and atherosclerosis [110]. Interestingly, although shorter (GT)n repeats typically enhance *HMOX1* expression, some studies suggest that increased levels of HO-1 associated with longer (GT)n alleles may correlate with an elevated risk of coronary heart disease and CKD [111].

#### 4.3.2. T(–413)A Single-Nucleotide Polymorphism

Another functional regulatory element within the *HMOX1* promoter region is the T(–413)A polymorphism (rs2071746), a single-nucleotide polymorphism. Experimental data suggest that the A allele enhances *HMOX1* gene transcription, thereby increasing its biological activity [110].

In clinical studies focused on diabetic kidney disease, the rs2071746 polymorphism has been shown to influence albuminuria occurrence. Specifically, individuals carrying the A allele presented a lower prevalence of albuminuria, whereas the TT genotype was associated with increased risk [112]. These findings suggest that the A allele may play a protective role in the early stages of DKD.

The functional relevance of *HMOX1* promoter polymorphisms has been broadly implicated in disease progression and susceptibility. Shorter (GT)n alleles contribute to increased *HMOX1* transcription, which may serve a protective role in kidney pathology through anti-inflammatory and antioxidative mechanisms [113]. However, dysregulation—whether due to excessively high or insufficient *HMOX1* expression—may contribute to disease development depending on the context and tissue environment [111,113].

## 5. New Markers in the Diagnosis of Diabetic Kidney Disease and Their Relationships with Fetuin-A and HO-1

The scientific community is increasingly discussing the need to develop methods for determining new biomarkers for use in the diagnosis of DKD. The most frequently mentioned are neutrophil gelatinase-associated lipocalin (NGAL), kidney injury molecule-1 (KIM-1), α-Klotho, microRNA and liver-type fatty acid binding protein (L-FABP). It seems interesting whether there are mechanisms linking well-known parameters with other potential biomarkers, which are the subject of this manuscript.

Currently, no direct association has been demonstrated between most biomarkers (NGAL, KIM-1, α-Klotho, L-FABP) and changes in fetuin-A levels in renal patients. No common mechanisms or molecular pathways linking these parameters are known. However, numerous studies provide evidence of correlations between these proteins [114,115,116,117]. In turn, specific microRNAs (miR-27a-3p, miR-27b-3p) may be responsible for regulating *AHSG* expression and may modulate inflammatory processes, fibrosis, and the response to renal injury, which may contribute to changes in fetuin-A concentrations [118,119,120]. Table 3 presents the potential association of fetuin-A with the previously mentioned biomarkers.

In turn, HO-1 expression in the kidney is strongly induced by damaging factors: hypoxia, hemoproteins, cytokines, and ROS. HO-1 exerts cytoprotective, antioxidant, and anti-inflammatory functions, therefore its role in kidney disease is primarily to limit damage and modulate the responses of other biomarkers [113]. There are several mechanisms that link HO-1 and the biomarkers mentioned above [125,126]. A summary of these mechanisms is presented in Table 4.

While most of the studies investigating the association between HO-1 and the listed biomarkers have been performed in the context of renal disease in general, these findings are also of relevance to DKD. The underlying mechanisms regulated by HO-1—such as oxidative stress, inflammation, and endothelial dysfunction—are central drivers of DKD progression. Therefore, the reported interactions between HO-1 and biomarkers including NGAL, KIM-1, α-Klotho, microRNA or L-FABP are likely to reflect processes directly involved in the pathogenesis of DKD. Although direct evidence linking HO-1 and these biomarkers specifically in DKD patients remains limited, the overlap of pathogenic pathways strongly suggests that these associations have translational value for DKD. Future studies are needed to validate these relationships in well-characterized DKD cohorts.

It should be emphasized that the proposed relationships between fetuin-A or HO-1 and other biomarkers in the context of DKD remain largely speculative. While the biological plausibility of these associations is supported by shared pathogenic pathways such as oxidative stress, inflammation, vascular calcification, and metabolic dysregulation, direct mechanistic data and clinical validation in DKD cohorts are currently limited. Therefore, these observations should be regarded as hypothesis-generating and highlight the need for future studies aimed at confirming the role of fetuin-A and HO-1 as clinically relevant biomarkers in DKD.

To facilitate a better understanding of the mechanisms linking fetuin-A and HO-1 with the discussed biomarkers, we have included a concise description of each biomarker. This description outlines their biological function, relevance in the context of DKD, methods of measurement, current limitations, and future perspectives. Such an approach aims to provide a more comprehensive framework for interpreting the potential role of these biomarkers in DKD pathogenesis and their possible translational value.

### 5.1. Neutrophil Gelatinase-Associated Lipocalin (NGAL)

Neutrophil gelatinase-associated lipocalin (NGAL) is a low molecular weight glycoprotein that is stored primarily in specific granules of neutrophils but is also expressed at low levels in other human tissues [137,138,139,140]. NGAL is an important transport protein that plays a significant role in physiological processes in the body. It influences, among others, the regulation of inflammation, generation of immune response and maintenance of metabolic homeostasis [141].

NGAL is freely filtered by the glomeruli and reabsorbed in the proximal tubules. In the case of damage to the epithelial tubules, there is a marked increase in urinary NGAL concentration [137,138]. This glycoprotein is considered a promising biomarker of kidney disease, especially as an early biomarker of acute kidney injury [142]. Some studies have reported increased urinary NGAL concentrations in individuals with well-controlled diabetes and normoalbuminuria [143]. However, these findings are based on cross-sectional data and therefore do not confirm the predictive role of NGAL in the development of DKD. Furthermore, NGAL levels may be elevated in other pathological conditions, such as hypertension, obesity, cardiovascular diseases, or metabolic complications such as insulin resistance, which limits the specificity of this marker [144].

Early kidney damage can be detected primarily by assessing plasma and urine NGAL levels, albuminuria and estimated glomerular filtration rate [144]. The urine NGAL to creatinine ratio has become useful in differentiating DKD from nondiabetic kidney disease [145]. Many studies also indicate a positive correlation of NGAL with albuminuria and renal tubular markers (RBP4, cystatin C, KIM-1) and a negative correlation with eGFR, which indicates the importance of NGAL in the progression of DKD [146,147,148].

NGAL can be determined by various methods, including enzyme-linked immunosorbent assay (ELISA), chemiluminescent microparticle immunoassay (CMIA), and radioimmunoassay (RIA). A lateral flow immunoassay (LFIA) has also been developed, which is a quick and simple tool for detecting NGAL as a point of care test (POCT) [149].

The limitations of NGAL use in DKD may be mainly due to the fact that many studies have shown increased levels of NGAL protein also in pathological conditions such as inflammation, atherosclerosis, and sepsis [150]. Increased systemic NGAL levels have also been observed in patients with chronic myeloid leukemia [151]. This may cause fluctuations in systemic and urinary NGAL levels and limit the use of NGAL as an accurate marker of renal damage [150]. Analytical factors such as biological variability or analytical performance of reagents and tolerance to NGAL interference should also be taken into account [150].

The meta-analysis conducted by Haase et al. [152] reports that the diagnostic cut-off for plasma NGAL in critically ill patients is 155.0 μg/L, with an AUC of 0.728. The prognostic cut-off is 179.2 μg/L (95% CI: 153.9–199.3), with an AUC of 0.775 (95% CI: 0.679–0.869). Other studies have reported a higher diagnostic cut-off value with a comparable AUC (234 μg/L, AUC = 0.71), and a lower prognostic cut-off value with a lower AUC (118 μg/L, AUC = 0.59). The authors suggest that these differences may be attributable to the heterogeneity of the study populations. The high heterogeneity of NGAL, particularly among intensive care unit patients, may lead to false-negative or false-positive results. Plasma NGAL levels were higher in patients with AKI compared to those without AKI; however, substantial inter-individual variability resulted in false-negative results in 56% of patients with AKI and false-positive results in 32% of patients without AKI. This variability complicates the use of a uniform diagnostic threshold in the general population [153].

In the future, further studies should be conducted focusing on the kinetics of systemic and urinary NGAL in various disease entities. In the case of disease states that may interfere with the accuracy of NGAL measurement, combining this protein with other biomarkers could increase the accuracy of prediction and risk stratification of kidney damage, but this requires further studies [150,151,154,155,156,157,158].

### 5.2. Kidney Injury Molecule-1 (KIM-1)

Kidney injury molecule-1 (KIM-1) is a transmembrane glycoprotein expressed in proximal tubules [159]. The normal reference level of KIM-1 in serum does not exceed 1 ng/mL [160]. KIM-1 is a marker of differentiation and proliferation, so it is practically undetectable in properly functioning kidneys, and its concentration increases after damage to the renal tubules [161,162]. According to studies, KIM-1 is a sensitive and specific marker of kidney damage [163]. One of the studies showed that urinary KIM-1 levels were significantly higher in patients with DKD than in patients with diabetes but without DKD. It was also noted that KIM-1 levels positively correlated with 24 h urinary albumin loss [164]. The AUC of uKIM-1 in patients with type 2 diabetes and normoalbuminuria was 0.85 (95% CI; 0.82–0.88) [165]. Another study showed a positive correlation between KIM-1 levels and the duration of diabetes, which could confirm the usefulness of KIM-1 as a marker in the early diagnosis of DKD [166]. KIM-1 levels can be measured in both serum and urine. The most commonly used method is ELISA [166,167].

The limitations of KIM-1 use are mainly due to the fact that most studies were conducted on small, limited numbers of patients. Some studies did not compare urinary KIM-1 concentrations with serum KIM-1 and did not take into account environmental and lifestyle characteristics of the patients that could have altered disease progression [166].

KIM-1 shows potential as a marker in the early diagnosis of DKD but it also has some limitations and requires further studies to better understand its prognostic value.

### 5.3. α-Klotho

α-Klotho is a transmembrane protein consisting of two domains: K1 and K2, which exhibit sequence homology with β-glycosidases. It occurs in three isoforms: a transmembrane form, a soluble form containing K1 and K2, and a truncated soluble form containing the K1 domain. Soluble Klotho is found in blood, urine, and cerebrospinal fluid [168].

Studies demonstrate that low Klotho concentrations may indicate renal dysfunction associated with diabetes [169]. The expression of Klotho in plasma and urine has also been shown to be reduced in the early stages of DKD and its further decrease may suggest the development of the disease [170,171,172,173]. In the case of diabetics, low levels of Klotho in serum have been correlated with a faster decline in eGFR. They also showed a negative correlation with albuminuria, in other words, reduced Klotho levels in the blood were associated with increased albuminuria [169,174,175,176,177]. Plasma and urine α-Klotho concentrations can be determined using human soluble α-Klotho immunoassay kits.

a-Klotho threshold values depend on the population, measurement method, and study objective. One study established a threshold value of 516.9 pq/mL for the diagnosis and development of CKD, with a sensitivity of 0.778 and a specificity of 0.705 [178]. Serum levels of this marker are significantly reduced in patients with CKD; in people with stages 3–4 of CKD, these concentrations are approximately 30–50% lower than in healthy individuals [177]. The level of this marker in serum showed a positive correlation with eGFR and a negative correlation with the occurrence of CKD, especially in the elderly, obese and diabetic patients, obese and elderly [176].

The limitations of using α-Klotho as a marker of DKD may be due to the lack of standardization of measurement methods and interactions between Klotho and various signalling pathways, e.g., mammalian target of rapamycin kinase (mTOR), peroxisome proliferator-activated receptor gamma (PPAR-γ), NF-κB that may affect Klotho concentration [179,180].

### 5.4. MicroRNA

MicroRNAs are a group of small (approximately 22 nucleotides), non-coding sequences that act as mediators of the post-transcriptional feedback control mechanism and participate in the regulation of inflammation [181,182,183,184].

MicroRNA expression is deregulated in pathological conditions occurring in chronic kidney diseases. The results of studies show that with the progression of kidney disease and a decrease in glomerular filtration rate, serum microRNA decreases. In advanced renal failure, reduced levels of miRNA-16, miRNA-21, miRNA-155, miRNA-210, miRNA-638 were found. It was also noted that in the case of advanced stages of chronic kidney disease, miRNA-125b, miRNA-145 and miRNA-155 decrease [185,186,187]. MicroRNAs, such as: miR-15b, miR-34a, miR-636, miR-21, miR-27a or miR-146a, are associated with the development of complications arising due to diabetes and may become biomarkers used to control the progression of the disease [188,189]. Studies have also confirmed that the expression of urinary exosomal miRNA (miR-320c, miR-6068, miR-1234-5p, miR-6133, miR-4270, miR-4739, miR-371b-5p, miR-638, miR-572, miR-1227-5p, miR-6126, miR-1915-5p, miR-4778-5p i miR-2861, miR-30d-5p, miR-30e-5p) in patients with DKD is changed [190].

MicroRNA expression can be detected in tissue samples and cell-free biological fluids (e.g., serum, plasma). Methods used to detect microRNA include quantitative polymerase chain reaction (qPCR), in situ hybridization, RNA sequencing, and microarrays [181].

Many studies have been performed to assess the level of microRNA in serum and urine of patients with type 1 and 2 diabetes in different stages of DKD, however the small number of patients proved to be a major limitation [50]. The inconsistent results obtained also resulted from different study designs and different types of analyzed microRNAs, therefore further validation studies would be necessary [191].

### 5.5. Liver-Type Fatty Acid Binding Protein (L-FABP)

Liver-type fatty acid binding protein (L-FABP) is an intracellular protein expressed mainly in the kidneys and liver [192]. The glomerulus filters the circulating portion of L-FABP, and then the protein is reabsorbed by the proximal tubules, which is why its increased concentration in urine is observed in proximal tubular cells damage [193]. Its increased expression also correlates with local and systemic inflammation [194]. It is increasingly being pointed out that urinary L-FABP excretion is associated with the protein/creatinine ratio (PCR), correlating with the onset and progression of DKD [195]. According to studies, urinary L-FABP concentration was significantly higher in patients with T2D who had normal urinary albumin concentration compared to normal control group in the case of renal failure. Therefore, urinary L-FABP concentration allows for detection of renal disease in patients with diabetes earlier than urinary albumin concentration [196]. Moreover, studies have been conducted in patients with type 1 diabetes, according to which urinary L-FABP is one of the independent predictors of renal tubular damage in DKD [192,197].

Diagnostic thresholds for L-FABP may vary depending on the study population and clinical context. For CKD monitoring, the cut-off value for urinary L-FABP is 17.4 µg/g creatinine [198], while for healthy individuals it is 8.4 µg/g creatinine [199]. The analysis of predictive performance showed that uL-FABP had an AUC of 0.623, whereas uPCR achieved an AUC of 0.706, indicating that uL-FABP demonstrates moderate accuracy in predicting disease progression [200]. L-FABP is most commonly measured using ELISA [196,200] but some studies also use latex-enhanced immunoturbidimetric technique [192]. However, full evaluation of the prognostic value of L-FABP is hampered by the lack of repeatable measurements of this protein, as indicated by some studies [200].

L-FABP shows potential as a marker for early detection and monitoring of progression of DKD, but additional studies should be conducted to assess its superiority over standard markers and the influence of other factors such as drugs.

**Figure 6:**
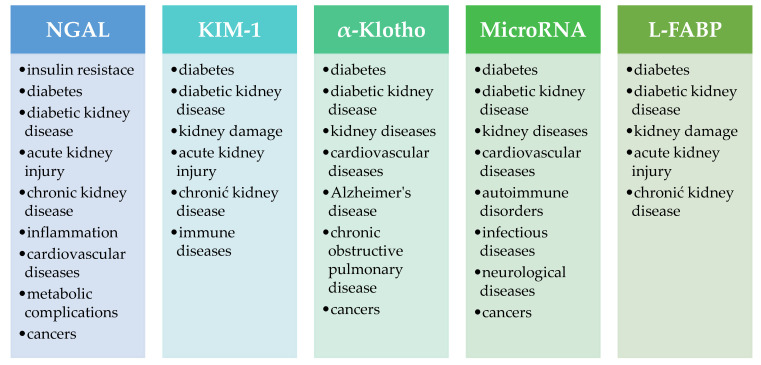
Examples of pathological conditions in which NGAL, KIM-1, α-Klotho, microRNA and L-FABP can be used as biomarkers [144,150,201,202,203,204,205]. NGAL—neutrophil gelatinase-associated lipocalin, KIM-1—kidney injury molecule-1, L-FABP—liver-type fatty acid binding protein.

Figure 6 presents examples of pathological conditions in which NGAL, KIM-1, α-Klotho, microRNA and L-FABP can be used as biomarkers.

### 5.6. The Influence of Comorbidities, Medications and Lifestyle Factors on the Concentrations of NGAL, KIM-1, α-Klotho, MicroRNA and L-FABP Biomarkers

The levels of biomarkers such as NGAL, KIM-1, α-Klotho, microRNA, and L-FABP may be influenced by a variety of clinical and environmental factors. Their concentrations can be influenced by various comorbidities and pathological conditions, such as inflammation, cardiovascular disease, diabetes, kidney damage, neurodegenerative diseases, and immunological disorders. Pharmacotherapy (e.g., RAA inhibitors) and lifestyle factors (exercise, diet, smoking) can also cause changes in marker levels, which is crucial for the correct interpretation of marker values and assessment of their clinical utility. Table 5 presents the of comorbidities, medications, and lifestyle factors that may affect the concentrations of DKD biomarkers.

## 6. Conclusions

Fetuin-A and HO-1 are well-known proteins that perform numerous functions in the human body and participate in numerous metabolic processes. However, disturbances in their synthesis and changes in their concentrations or activity can lead to the development of diseases. AHSG polymorphisms have been shown to be involved in the pathophysiology of obesity, insulin resistance, diabetes, cardiovascular disease, and kidney disease, including diabetic kidney disease. HMOX1 polymorphisms, in turn, may play a role in the development and progression of coronary heart disease and chronic kidney disease.

As the examples above demonstrate, fetuin-A, HO-1, and genetic variations associated with the genes encoding these proteins may be involved in the development of many metabolic diseases, including lifestyle diseases such as diabetes and its complications. Currently, there is an urgent need to develop rapid and effective algorithms for diagnosing these conditions. This is particularly important in the case of DKD, as traditional biomarkers used to diagnose this disease only change when the kidneys are already severely damaged. For these reasons, it is crucial to focus on identifying and developing new parameters that could improve the diagnostic process and thus contribute to faster implementation of appropriate treatment.

In summary, both fetuin-A and HO-1 represent promising biomarkers with potential clinical relevance in nephrology. Fetuin-A, through its association with inflammatory processes, insulin resistance, and cardiovascular risk, may in the future be applied in identifying metabolically burdened patients as well as in monitoring treatment efficacy in individuals with chronic kidney disease. HO-1, as an enzyme with cytoprotective and antioxidant properties, may serve both as a marker of oxidative stress accompanying kidney disease progression and as a potential therapeutic target in conditions with inflammatory and metabolic backgrounds. Future research should focus on prospective validation of these biomarkers in large nephrology cohorts, evaluation of their prognostic value, and the development of diagnostic panels integrating fetuin-A and HO-1 with other markers of kidney injury and function. Such an approach could, in the future, contribute to earlier detection of kidney diseases and to more personalized therapeutic strategies in clinical practice.

## Figures and Tables

**Table 1:** Classification of urinary albumin concentration according to the KDIGO 2024 Clinical Practice Guideline for the Evaluation and Management of Chronic Kidney Disease [52,53].

	Albuminuria (ACR) Categories		
	A1 Normal to Mildly Increased	A2 Moderately Increased	A3 Severely Increased
**Albumin excretion rate (mg/24 h)**	<30	30–300	>300
**Albumin/creatinine ratio (mg/g)**	<30	30–300	>300
**Albumin/creatinine ratio (mg/mmol)**	<3	3–30	>30

**Table 2:** The comparison of cystatin C and creatinine [62,63].

	Cystatin C	Creatinine
**Advantages**	- endogenous substance rises faster than creatinine not dependent on muscle mass, physical activity or protein intake	endogenous substance low cost low intra-individual variability
**Factors influencing** **concentration**	ethnicity thyroid dysfunction medications (corticosteroids, cyclosporine)	age gender physical activity muscle mass medications (cimetidine) diet

**Cystatin C**

**Creatinine**

**Table 3:** The relationship between fetuin-A and selected biomarkers in the context of kidney diseases.

Biomarker	Importance in Kidney Diseases	Mechanisms Linking with Fetuin-A	References
NGAL	Parallel changes in acute kidney injury (AKI): NGAL increases in AKI, fetuin-A may decrease in inflammation/severe AKI/CKD.	Both NGAL and fetuin-A respond to inflammation and tubular damage; IL-6 and other cytokines can decrease fetuin-A and simultaneously increase NGAL.	[5,115,121]
KIM-1	KIM-1 increases in proximal tubule injury; may correlate with fetuin-A in AKI/CKD.	KIM-1 and fetuin-A share a common context of renal injury and inflammation; perhaps the decrease in fetuin-A level is associated with increased markers of tubular damage.	[114,122]
α-Klotho	Both proteins are associated with disturbances in mineral homeostasis and vascular calcification. Clinical studies have observed co-dysregulation of these proteins in advanced CKD.	Common pathways regulating phosphorus/calcium homeostasis and processes leading to vascular calcification; fetuin-A acts as a calcification inhibitor and α-Klotho has a role in mineral metabolism—together influence the risk of calcification and CKD progression.	[116,123]
MicroRNA	MicroRNA can regulate genes associated with inflammation, fibrosis, and markers of kidney damage.	MicroRNAs (miR-27a-3p, miR-27b-3p) can modulate the expression of *AHSG* and other genes (profibrotic, inflammatory, and injury markers). This suggests a possible direct association of microRNAs with fetuin-A.	[118,119,120]
L-FABP	L-FABP is used as a marker of nephron damage; in kidney diseases, correlations between markers of damage and liver metabolic markers (such as fetuin-A) are often observed.	L-FABP and fetuin-A levels may change in parallel in response to renal injury and metabolic disturbances. There is no clear evidence of a regulatory relationship.	[117,124]

AKI—acute kidney injury; CKD—chronic kidney disease; NGAL—neutrophil gelatinase-associated lipocalin; KIM-1—kidney injury molecule-1; L-FABP—liver-type fatty acid binding protein.

**Table 4:** The relationship between HO-1 and selected biomarkers in the context of kidney diseases.

Biomarker	Importance in Kidney Diseases	Mechanisms Linking with HO-1	References
NGAL	NGAL levels increases in AKI, and HO-1 is strongly induced by oxidative stress and hemoproteins in AKI. In animal models, early induction of HO-1 can reduce NGAL expression in the later phase of injury.	Both NGAL and HO-1 participate in the regulation of iron metabolism. HO-1 degrades heme into bilirubin, CO, and iron, and also reduces oxidative stress. NGAL is induced by, among other factors, oxidative stress damage and free iron.	[127,128]
KIM-1	KIM-1 levels are increased in AKI. Preclinical studies have shown that induction of HO-1 reduces tubular damage and downregulates KIM-1 expression.	The anti-inflammatory and antioxidant effects of HO-1 limit the activation of proximal tubular cells, which may secondarily reduce KIM-1 expression.	[125,129]
α-Klotho	α-Klotho levels are decreased in CKD. Klotho may promote the kidney transcription of HO-1.	HO-1 and α-Klotho reduce oxidative stress and inhibit fibrosis. Furthermore, α-Klotho can be induced by the products of the HO-1-mediated reaction.	[130,131,132]
MicroRNA	Several miRNAs (miR-217, miR-377, miR-155) regulate HO-1 expression. In turn, HO-1 influences the miRNA profile in renal cells. Some HO-1-related miRNAs participate in the regulation of markers of renal injury.	miRNAs control HO-1 translation, and HO-1 can modulate miRNA expression by reducing oxidative stress and altering the activity of transcription factors (Nrf2).	[133,134]
L-FABP	Oxidative stress increases L-FABP levels. In models of nephrotoxicity, induction of HO-1 reduces damage and lowers L-FABP levels.	HO-1 reduces ROS generation and lipid peroxidation, which reduces L-FABP release.	[135,136]

HO-1—heme oxygenase 1; AKI—acute kidney injury; CKD—chronic kidney disease; NGAL—neutrophil gelatinase-associated lipocalin; KIM-1—kidney injury molecule-1; L-FABP—liver-type fatty acid binding protein.

**Table 5:** Examples of comorbidities, medications, and lifestyle factors that may affect the concentrations of DKD biomarkers.

Biomarker	Examples of Comorbidities That May Affect the Concentrations of Biomarkers	Examples of Medications That May Affect the Concentrations of Biomarkers	Examples of Lifestyle Factors That May Affect the Concentrations of Biomarkers	References
NGAL	Inflammation Arterial hypertension Obesity Diabetes Metabolic complications Cancers Cardiovascular diseases Sepsis	Atorvastatin (lowers plasma NGAL levels)	Exercise Diet	[144,150,206,207]
KIM-1	Immune diseases (asthma, allergies, ectopic dermatitis, rheumatoid arthritis, systemic lupus erythematosus) Diabetes Kidney damage	Dapagliflozin treatment (significantly reduces KIM-1 levels)	Exercise (high-intensity interval training)	[122,201,208]
α-Klotho	Renal failure Diabetes Neurodegenerative diseases Cardiovascular diseases Chronic obstructive pulmonary disease Cancers	Renin–angiotensin system inhibitors (losartan, valsartan) Statin (fluvastatin) mTOR inhibitors (rapamycin, everolimus) Vitamin D and pentoxifylline Supplements and traditional medicines (astaxanthin, cordycepin, curcumin, resveratrol, ligustilide)	Exercise and sport activity Ageing	[202]
MicroRNA	Diabetes Kidney diseases Cardiovascular diseases Autoimmune disorders Infectious diseases Neurological diseases Cancers	Epigallocatechin-3-gallate (EGCG) and quercetin (upregulate miR-29)	Exercise Diet Smoking	[203,204,209,210,211]
L-FABP	Diabetes Kidney damage Acute kidney injury Chronic kidney disease	Combination therapy with the ARB olmesartan and the ACE inhibitor temocapril (significantly reduces elevated urinary L-FABP levels)	Sport activity	[198,205,206]

## Data Availability

Not applicable.

## Abbreviations

The following abbreviations are used in this manuscript:

DKD	Diabetic kidney disease
DN	Diabetic nephropathy
ESRD	End-stage renal disease
HO-1	Heme oxygenase 1
*AHSG*	Gene encoding fetuin-A
Ang-II	Angiotensin II
TGF-β	Transforming growth factor-β
ROS	Reactive oxygen species
NADPH	Reduced nicotinamide adenine dinucleotide phosphate
RAS	Renin–angiotensin system
NF-κB	Nuclear factor kappa B
GFR	Glomerular filtration rate
KDIGO	Kidney Disease: Improving Global Outcomes
T2D	Type 2 diabetes
TNF-α	Tumour necrosis factor α
eGFR	Estimated glomerular filtration rate
IFN-γ	Interferon gamma
SS	Signal sequence
CP	Connecting peptide
FFA	Free fatty acids
SNPs	Single-nucleotide polymorphisms
CAC	Coronary artery calcification
CKD	Chronic kidney disease
CRP	C-reactive protein
CAD	Coronary artery disease
CACS	Coronary artery calcification score
GDM	Gestational diabetes mellitus
CO	Carbon monoxide
Fe^2+^	Free ferrous iron
O_2_	Molecular oxygen
APC	Antigen-presenting cells
SMCs	Smooth muscle cells
COPD	Chronic obstructive pulmonary disease
UV	Ultraviolet
CoPP	Cobalt protoporphyrin IX
Nrf2	Nuclear factor erythroid 2–related factor 2
MAPKs	Mitogen-activated protein kinases
PKC	Protein kinase C
AMPK	AMP-activated protein kinase
PI3K/Akt	Phosphoinositide 3-kinase/protein kinase B
AP-1	Activator protein-1
STAT3	Signal transducer and activator of transcription 3
YY1	Yin Yang 1
HIF-1	Hypoxia-inducible factor 1
ZnPPIX	Zinc protoporphyrin IX
SnPPIX	tin protoporphyrin IX
LPS	Lipopolysaccharide
Keap1	Kelch-like ECH-associated protein 1
PAMPs	Pathogen-associated molecular patterns
PI3K	Phosphatidylinositol 3-kinase
Bach1	BTB and CNC homology 1
NGAL	Neutrophil gelatinase-associated lipocalin
ELISA	Enzyme-linked immunosorbent assay
CMIA	Chemiluminescent microparticle immunoassay
RIA	Radioimmunoassay
LFIA	Lateral flow immunoassay
POCT	Point of care test
KIM-1	Kidney injury molecule-1
mTOR	Mammalian target of rapamycin kinase
PPAR-γ	Peroxisome proliferator-activated receptor gamma
qPCR	Quantitative polymerase chain reaction
L-FABP	Liver-type fatty acid binding protein
PCR	Protein/creatinine ratio
ACE	Angiotensin-converting enzyme
AKI	Acute kidney injury
